# Abnormal migration behavior linked to Rac1 signaling contributes to primordial germ cell exhaustion in Fanconi anemia pathway-deficient *Fancg*^−/−^ embryos

**DOI:** 10.1093/hmg/ddab222

**Published:** 2021-08-09

**Authors:** Amandine Jarysta, Lydia Riou, Virginie Firlej, Clémentine Lapoujade, Thierry Kortulewski, Vilma Barroca, Anne-Sophie Gille, Florent Dumont, Sébastien Jacques, Franck Letourneur, Filippo Rosselli, Isabelle Allemand, Pierre Fouchet

**Affiliations:** Université de Paris and Université Paris-Saclay, iRCM/IBFJ CEA, UMR Stabilité Génétique Cellules Souches et Radiations, Laboratoire des Cellules Souches Germinales, Fontenay-aux-Roses F-92265, France; Université de Paris and Université Paris-Saclay, iRCM/IBFJ CEA, UMR Stabilité Génétique Cellules Souches et Radiations, Laboratoire des Cellules Souches Germinales, Fontenay-aux-Roses F-92265, France; Université de Paris and Université Paris-Saclay, iRCM/IBFJ CEA, UMR Stabilité Génétique Cellules Souches et Radiations, Laboratoire des Cellules Souches Germinales, Fontenay-aux-Roses F-92265, France; Université de Paris and Université Paris-Saclay, iRCM/IBFJ CEA, UMR Stabilité Génétique Cellules Souches et Radiations, Laboratoire des Cellules Souches Germinales, Fontenay-aux-Roses F-92265, France; Université de Paris and Université Paris-Saclay, Inserm, iRCM/IBFJ CEA, UMR Stabilité Génétique Cellules Souches et Radiations, Laboratoire de RadioPathologie, Fontenay-aux-Roses F-92265, France; Université de Paris and Université Paris-Saclay, Inserm, iRCM/IBFJ CEA, UMR Stabilité Génétique Cellules Souches et Radiations, Fontenay-aux-Roses F-92265, France; Université de Paris and Université Paris-Saclay, iRCM/IBFJ CEA, UMR Stabilité Génétique Cellules Souches et Radiations, Laboratoire des Cellules Souches Germinales, Fontenay-aux-Roses F-92265, France; Département de Génétique, Développement et Cancer. Team From Gametes to Birth, Institut Cochin, INSERM U1016, Paris F-75014, France; Université Paris Saclay, UMS IPSIT, Châtenay-Malabry F-92296, France; Plate-Forme Séquençage et Génomique, Institut Cochin, Inserm U1016, Université de Paris, 22 rue Méchain Paris 75014, France; Plate-Forme Séquençage et Génomique, Institut Cochin, Inserm U1016, Université de Paris, 22 rue Méchain Paris 75014, France; CNRS-UMR9019, Intégrité du Génome et Cancers, Université Paris-Saclay, Villejuif 94805, France; Université de Paris and Université Paris-Saclay, iRCM/IBFJ CEA, UMR Stabilité Génétique Cellules Souches et Radiations, Laboratoire des Cellules Souches Germinales, Fontenay-aux-Roses F-92265, France; Université de Paris and Université Paris-Saclay, iRCM/IBFJ CEA, UMR Stabilité Génétique Cellules Souches et Radiations, Laboratoire des Cellules Souches Germinales, Fontenay-aux-Roses F-92265, France

## Abstract

Fanconi anemia (FA) is a rare human genetic disorder characterized by bone marrow failure, predisposition to cancer and developmental defects including hypogonadism. Reproductive defects leading to germ cell aplasia are the most consistent phenotypes seen in FA mouse models. We examined the role of the nuclear FA core complex gene *Fancg* in the development of primordial germ cells (PGCs), the embryonic precursors of adult gametes, during fetal development. PGC maintenance was severely impaired in *Fancg*^−/−^ embryos. We observed a defect in the number of PGCs starting at E9.5 and a strong attrition at E11.5 and E13.5. Remarkably, we observed a mosaic pattern reflecting a portion of testicular cords devoid of PGCs in E13.5 fetal gonads. Our *in vitro* and *in vivo* data highlight a potential role of *Fancg* in the proliferation and in the intrinsic cell motility abilities of PGCs. The random migratory process is abnormally activated in *Fancg*^−/−^ PGCs, altering the migration of cells. Increased cell death and PGC attrition observed in E11.5 *Fancg*^−/−^ embryos are features consistent with delayed migration of PGCs along the migratory pathway to the genital ridges. Moreover, we show that an inhibitor of RAC1 mitigates the abnormal migratory pattern observed in *Fancg*^−/−^ PGCs.

## Introduction

Fanconi anemia (FA) is a recessive disease characterized by congenital defects, progressive bone marrow (BM) failure and predisposition to cancer, including acute myeloid leukemia and squamous cell carcinoma. In total, 21 genes (*FANCA, FANCB, FANCC, FANCD1/BRCA2, FANCD2, FANCE, FANCF, FANCG/XRCC9, FANCI, FANCJ/BRIP1/BACH1, FANCL/PHF9/Pog, FANCM/Hef, FANCN/PALB2, FANCO/Rad51c, FANCP/SLX4, FANCQ/XPF, FANCR/RAD51, FANCS/BRCA1, FANCT/UBE2T*, *FANCU/XRCC2* and *FANCV/REV7*) along with FA Associated Proteins FAAP100, FAAP20 and FAAP24 have been identified to participate in the emergence of FA pathology (1). The FA pathway is involved in DNA replication, DNA repair and cell cycle checkpoints. In response to a stalled replication fork or to DNA damage, a group of eight proteins (FANCA, B, C, E, F, G, M and L) forms a nuclear core complex. Then, via the E3-ligase activity of FANCL, this complex mediates the mono-ubiquitination of a second group of proteins composed of FANCD2 and FANCI (the ID complex). Once mono-ubiquitinated, the ID complex is recruited to DNA repair foci and interacts with a third group of FANC proteins (FANCD1, FANCJ, FANCN, FANCP and FANCS) and other BRCA1-interacting proteins. The *FANCA, FANCC* and *FANCG* components of the core complex are responsible for nearly 90% of FA cases, and the loss of any core complex component impairs ubiquitination of the ID complex (1,2). FANCG/XRCC9 is a 65 kDa protein with at least seven protein–protein interaction tetratricopeptide repeat motifs (3).

Stem cells are responsible for the homeostasis of many tissues, whose pool is critically determined during embryonic life. The genomic integrity of stem cells is fundamental for the establishment of the stem cell pool, and some DNA repair abnormalities are linked to stem cell dysfunction (4). As FA is a DNA repair syndrome, the FA pathway is involved in neural and hematopoietic progenitor/stem cell failure during fetal and adult life (5–10). However, increased production of reactive oxygen species, abnormal activation of stress-activated protein kinase or TGFβ and enhanced sensitivity to endogenous aldehyde or cytokines such as tumor necrosis factor-alpha and interferon-gamma have also been reported to play a role in FA pathology (11–17).

Reproductive defects are the most consistent phenotypes seen in FA mouse models [*Fanca*^−/−^, *Fancb*^−/−^  *Fancc*^−/−^, *Fancd1, Fancd2*^−/−^, *Fance*^−/−^, *Fancg*^−/−^, *Fancj*^−/−^, *Fancl*^−/−^, *Fancm*^−/−^ and *Fancp*^−/−^ (18–22)]. In males, the size of the spermatogonial stem cell (SSCs) pool in adults results from a complex ontogenic process that begins during the embryonic period with the emergence and amplification of SSC precursors, the primordial germ cells [PGCs; (23)]. PGCs first emerge at the base of the incipient allantois at ~E6.5, then migrate toward the urogenital ridge along the epithelia of the hindgut and the mesentery between E8.5 and E10.5, and finally colonize the genital ridges at E11.5. Next, PGCs proliferate and differentiate in the primitive gonad according to sex-specific paths where germ cells in XX gonads enter the prophase of meiosis at E14.5, whereas those in XY gonads arrest in mitosis (24). Although *Fanca*, *Fancc*, *Fancj, Fancl*, *Fancm*, *Fancp* and *Fancv* genes have been shown to affect the number of PGCs in mouse embryos (20–22,25–28), the mechanisms responsible for the progressive depletion of germinal cells remain poorly understood. Several lines of evidence also suggest roles of *Fanca*, *Fancb, Fancd2, Fancj* and *Fancp* in male meiosis in adult testis (21,22,26,29,30).

Here, we showed that deletion of the gene *Fancg,* a member of the FA core complex, impairs the maintenance of PGCs during fetal development. We found that loss of *Fancg* resulted in a defect in the number of PGCs starting at E9.5 and a strong attrition at E11.5 and E13.5, along with a mosaic pattern of testicular cords in E13.5 fetal gonads with cords devoid of PGCs. Our data highlight a potential role of *Fancg* during the migration of PGCs from E8.5 to E11.5, allowing them to reach and aggregate in the genital ridges. PGC attrition in E11.5 *Fancg*^−/−^ embryos is consistent with delayed PGC migration, owing to an aberrant migrative behavior that could be modulated by an inhibitor of Rac1. Hence, we hypothesize that the progressive decrease in PGCs all along the migratory path up to E11.5 is linked both to an intrinsic proliferation defect of *Fancg*^−/−^ PGCs and to the combination of perturbed migration and subsequent cell death in delayed PGCs at E11.5.

## Results

### PGCs are substantially reduced in Fancg mutant mice

**Figure 1:**
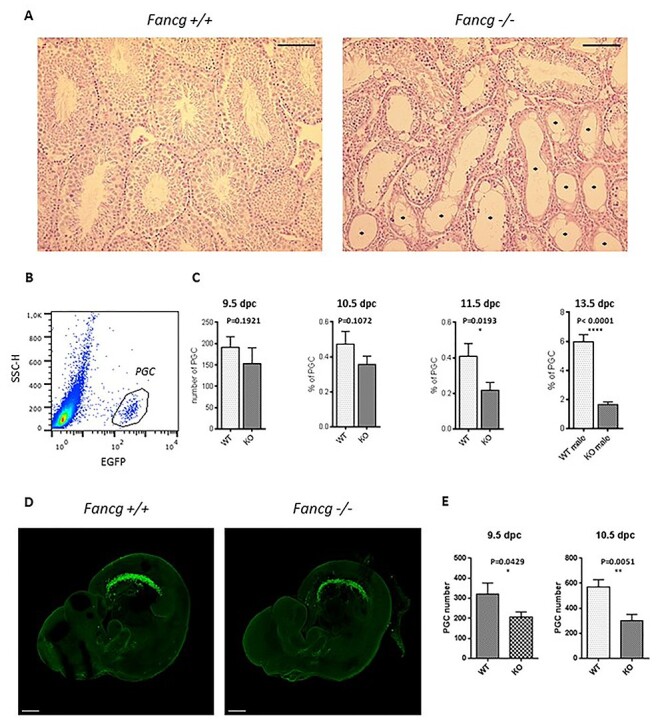
*Fancg*  ^−/−^ mice exhibit loss of germinal cells in embryos and adult testis. (**A**) Hematoxylin–eosin-stained histological sections of testes of adult 3-month-old *Fancg^+/+^* and *Fancg*^−/−^ mice. *Fancg*^−/−^ testis showed germinal aplasia with SCO tubules (^*^) (scale = 100 μm). (**B**) EGFP-positive PGC identification in E13.5 *OG2:Fancg^+/+^* embryos using flow cytometry. (**C**) Quantification of the absolute number of PGCs per embryo using Trucount microbeads at E9.5 dpc wild-type (WT) *n* = 21, knockout (KO) *n* = 10), of the frequency of PGCs per embryo at E10.5 (WT *n* = 17, KO *n* = 15), and of the frequency of PGCs per gonads at E11.5 (WT *n* = 12, KO *n* = 11) and E13.5 (WT *n* = 8, KO *n* = 9). (**D**) Confocal microscopy analysis of E10.5 cleared embryos using an antibody against EGFP to identify PGCs (scale = 300 μm). (**E**) Automated cell counting of PGCs per embryo using Imaris software at E9.5 (WT *n* = 4, KO *n* = 5) and E10.5 (WT *n* = 4, KO *n* = 5).

Histological analysis (Fig. 1A) revealed that, compared with the testes of wild-type (WT) controls, the testis of *Fancg*^−/−^ mice appeared atrophic with germ cell aplasia and displayed a mosaic pattern of seminiferous tubules consisting of tubules exhibiting normal spermatogenesis and agametic Sertoli-cell-only (SCO) tubules, as previously reported (31). Deficient prenatal germ cell development and PGC attrition were previously described in other Fanconi mouse models (19). We aimed to investigate the development of PGCs in *Fancg*^−/−^ mice, presuming a defect in the fetal germ cell lineage. For this purpose, *Fancg*^+/−^ mice were backcrossed with a transgenic OG2 mouse in which enhanced green fluorescent protein (EGFP) is controlled by the distal enhancer of the *Oct3/4* promoter to identify the embryonic stage at which germ cell depletion begins (32). This model allows PGCs to be tracked starting around E8.5 after their specification in the embryo and to follow PGC migration along the epithelia of the hindgut, PGC colonization of genital ridges at E11.5 and PGC proliferation and differentiation in the primitive gonads at least up to E14.5. First, we analyzed PGCs from *Fancg* mutant embryos at E9.5, E10.5, E11.5 and E13.5 using flow cytometry (Fig. 1B). The population of PGCs was substantially diminished in the bipotential gonads at E11.5 and in the developing fetal testis (Fig. 1C) and ovaries (Supplementary Material, Fig. S1A) at E13.5. The decay of PGCs at E11.5 and E13.5 was confirmed by histological analysis in an FVB background *Fancg*^−/−^ mice using germinal specific MVH marker (Supplementary Material, Fig. S1B and C). To refine the characterization of the defects in PGC development at E9.5 and E10.5, confocal analysis of embryos was performed to detect PGCs using anti-EGFP antibody after clearing and transparization of tissue by the 3DISCO protocol (Fig. 1D). This method enables entire embryos to be imaged without sectioning and a 3D tissue reconstruction. With this technique, we showed that the defect in the number of PGCs started at E9.5 and that the number of PGCs was severely affected when they began to migrate bilaterally to the gonads at E10.5 (Fig. 1E).

### Proliferation of PGCs is decreased in Fancg ^−/−^ embryos, and mutant PGCs show increased cell death

**Figure 2:**
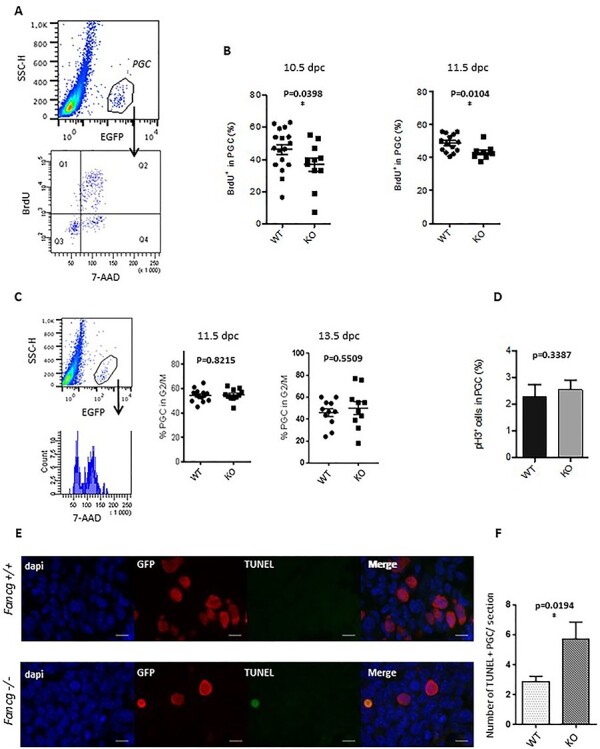
Decreased proliferation rate and increased cell death of PGCs in *Fancg*^−/−^ embryos at E11.5. (**A**) Identification of the proliferative BrdU-positive cell fraction (Q1 and Q2) in the EGFP-positive PGC population from E11.5 WT embryos. (**B**) Quantification of the proliferative fraction of PGCs at E10.5 (WT *n* = 17, KO = 11) and E11.5 (WT *n* = 14, KO = 15) in *OG2:Fancg^+/+^* and *OG2:Fancg*^−/−^ embryos. (**C**) DNA content analysis (7-AAD) of the EGFP-positive PGC population at E11.5 (WT *n* = 5, KO *n* = 4) and E13.5 (WT *n* = 11, KO *n* = 10). (**D**) Frequency of mitotic PHH3-positive PGCs counted from histological sections of testes in E13.5 *OG2:Fancg^+/+^* and *OG2:Fancg*^−/−^ embryos (WT *n* = 3, KO *n* = 3). (**E**) Detection of cell death by TUNEL assays in histological sections of E11.5 *OG2:Fancg^+/+^* and *OG2:Fancg*^−/−^ embryos. Nuclei counterstained by DAPI (blue), PGCs labeled with an antibody against EGFP (red) and the TUNEL-positive signal (green) are shown (scale = 10 μm). (**F**) Quantification of the number of TUNEL-positive dying cells per section at E11.5 (WT *n* = 6, KO *n* = 6).

Owing to the proliferating state of PGCs and the role FA pathway in the S and G2/M phase of the cell cycle, we investigated whether the decay in the number of PGCs in *Fancg*-deficient mice could result from altered PGC proliferation. The decay in the number of PGCs in *Fancg*-deficient mice could result from altered PGC proliferation. Indeed, PGCs rapidly proliferate during all stages of migration and after colonization of the gonads, and the FA pathway is known to play a role in the S and G2/M phases of the cell cycle. A BrdU incorporation assay was performed by flow cytometry of PGCs from male littermate embryos at E10.5 and E11.5 (Fig. 2A). After 2 h of BrdU incorporation, a small decrease in the percentage of BrdU-positive PGCs was observed in *Fancg*^−/−^ embryos at E10.5 and E11.5 compared with WT embryos, indicating a mild defect of the proliferation rate (Fig. 2B). However, we did not observe any G2/M blockade in *Fancg*^−/−^ PGCs (Fig. 2C), nor any changes in the proportion of PGCs positive for the mitotic cell marker phospho-histone H3 [PHH3; Fig. 2D and Supplementary Material, Fig. S2]. Hence, *Fancg* deficiency appears to affect entry into and progression of the S phase of PGCs.

To assess whether cell death contributes to the decrease of PGC numbers, fluorescent TUNEL analysis was realized in E11.5 embryos to detect dead cells. A significant increase in TUNEL-positive PGCs was detected in E11.5 *Fancg*^−/−^ embryonic gonads compared with littermate controls (Fig. 2E and F). Thus, the TUNEL analysis confirmed that cell death accounts for the dramatic loss of germ cells observed in *Fancg*^−/−^ embryos. Taken together, these observations suggested that defects in both PGC proliferation and survival during PGC development account for germ cell depletion in *Fancg*^−/−^ mice.

### The heterogeneous phenotype of testicular cords at E13.5 suggests a defect of genital ridge colonization by PGCs at E11.5

**Figure 3:**
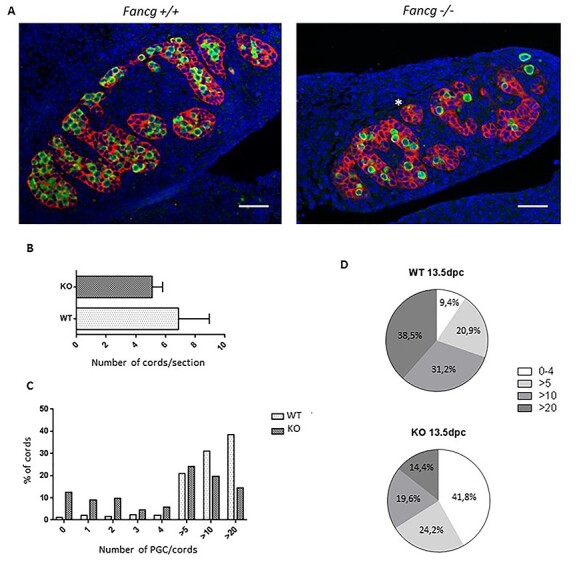
E13.5 testis show a mosaic pattern of testicular cords containing PGCs and cords devoid of PGCs. (**A**) Histological sections of E13.5 *OG2:Fancg^+/+^* and *OG2:Fancg*^−/−^ male embryos. PGCs are labeled with an antibody against EGFP (green), Sertoli cells are labeled with an antibody against AMH (red) and nucleus are stained with DAPI [blue; (scale = 50 μm)]. (**B**) Number of testicular cords per histological section in *OG2:Fancg^+/+^* and *OG2:Fancg*^−/−^ E13.5 embryos (WT *n* = 3, KO *n* = 3). (**C**) Repartition of testicular cords (WT *n* = 5, KO *n* = 5) as a function of the number of PGCs contained in each cord in E13.5 testis. (**D**) Pie chart summarizing the distribution of cords in E13.5 *OG2:Fancg^+/+^* and *OG2:Fancg*^−/−^ testis observed in (C).

We observed no gross differences in testicular cord formation between WT and knockout (KO) male fetal gonads. The number of cords in fetal testis was relatively similar in E13.5 WT and *Fancg*^−/−^ littermate embryos (Fig. 3A and B). In addition, the distributions of fetal anti-müllerian hormone (AMH)-positive Sertoli cells and 3βHSD Leydig cells appeared grossly similar between controls and *Fancg* mutants at E13.5 (Fig. 3A and Supplementary Material, Fig. S3), and thus, *Fancg* deficiency does not appear to alter testicular cord formation. However, we observed a mosaic pattern with a strong increase in the number of testicular cords devoid of PGCs in E13.5 testis (Fig. 3A and C). Approximately 40% of cords contained <4 PGCs in *Fancg* KO mice compared with <10% of cords in WT mice (Fig. 3D). The simultaneous observation of cords full of PGCs and cords devoid of PGCs at E13.5 appears incompatible with the global mild defect of proliferation observed in *Fancg* KO PGCs as the primary cause of PGC attrition. Indeed, a global proliferation deficiency would logically result in a homogenous decrease in the number of PGCs per cords. Hence, this mosaic pattern of testicular cords seems also to be linked to a primary defect of PGCs preventing them from successfully colonizing the genital ridges at E11.5 and resulting in poor clustering of PGCs coalescing with somatic cells to initiate germ cell expansion in developing cords. Thus, this mosaic pattern observed at E13.5 suggests that the defect in mutant mice is linked to an impairment in the first phase of PGC development (up to E11.5) during which PGCs migrate and proliferate.

### Fancg ^−/−^ PGCs show abnormal migration properties

**Figure 4:**
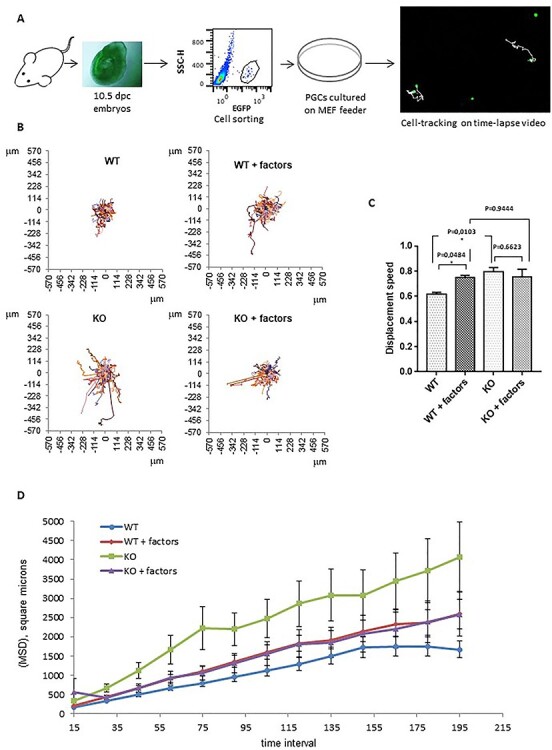
E10.5 *OG2:Fancg*^−/−^ PGCs display *in vitro* abnormal migration behavior. (**A**) Summary of the *in vitro* migration assay of sorted PGCs from E10.5 embryos tracked by EGFP fluorescence (acquisition over 7.5 h, every 15 min). (**B**) Movements of *OG2:Fancg^+/+^* and *OG2:Fancg*^−/−^ PGCs without and with migration factors (KITL, SDF-1, BMP4 and LIF) shown from the same origin point. (**C**) Average speed and (**D**) MSD of the *OG2:Fancg^+/+^* and *OG2:Fancg*^−/−^ PGCs with or without the addition of factors to the medium (WT vs WT + factors, *P* = 0.0027; WT vs KO, *P* = 0.0005).

The germinal defects observed in *Fancg*^−/−^ embryos occurred during a stage of intense migration of PGCs. As we observed an increased number of apoptotic PGCs at E11.5 in mutant mice and given that the fate of PGCs failing to reach the gonads is death (33), we investigated the migration behaviors of PGCs. We developed *in vitro* assays to study the migration competence of PGCs. Directional migration allows cells to rapidly move between points, whereas random migration allows cells to explore their local environment. Both the speed and the directionality of cell motility regulate migration. Cells migrating more randomly are characterized by decreased directional migration. Using a feeder 2D substrate system in which PGCs were sorted from a pool of E10.5 embryos by flow cytometry and seeded on inactivated mouse embryo fibroblasts (MEFs; nearly 300 PGCs/well), the random migration of PGCs was analyzed by time lapse video microscopy to capture the motility responses of individual PGCs (Fig. 4A and B, and Supplementary Material, video 1). Cell speed and mean square displacement (MSD) are migration parameters calculated from the cell tracking data. MSD is related to the territory explored by a cell (34). An elevated cell speed (Fig. 4C) and a higher MSD (Fig. 4D) illustrated that WT PGCs explored a larger territory in the presence of cytokines and growth factors than in control conditions. It was expected as kit ligand (KITL), stromal-derived factor 1 (SDF-1), leukemia inhibitory factor (LIF) and bone morphogenetic protein 4 (BMP4) factors are known to stimulate PGC maintenance and migratory competence (35,36). Increased exploration was mainly because of higher cell speed rather than increased directional persistence, displayed as the ratio of the distance between two points to the distance of the actual trajectory (directionality ratio) or as the direction autocorrelation function (Supplementary Material, Fig. S4A and A′). First, these data on WT PGCs in the presence of factors known to stimulate their migratory competence validated our analytical approach. In basal medium conditions, *Fancg*^−/−^ PGCs explored a wider territory than WT PGCs, as shown by cell tracks (Fig. 4B), owing to increased cell speed (Fig. 4C) but not to directional persistence (Supplementary Material, Fig. S4A and A′). Indeed, the speed of *Fancg*^−/−^ PGCs in basal conditions was comparable with the speed of stimulated WT PGCs in the presence of factors (Fig. 4C), and the MSD of *Fancg*^−/−^ PGCs was highly increased (Fig. 4D, *P* = 0.0005). Moreover, *Fancg*^−/−^ PGCs did not respond to the stimuli by KITL, SDF-1, LIF or BMP4, as cell speed did not vary after stimulation (Fig. 4C). Likewise, the MSD of *Fancg*^−/−^ PGCs did not respond normally when stimulated by these factors (Fig. 4D, *P* = 0.1854). This result suggests an abnormal activation of the random migratory process in *Fancg*^−/−^ PGCs, altering the migration of cells in response to varied extracellular stimuli.

**Figure 5:**
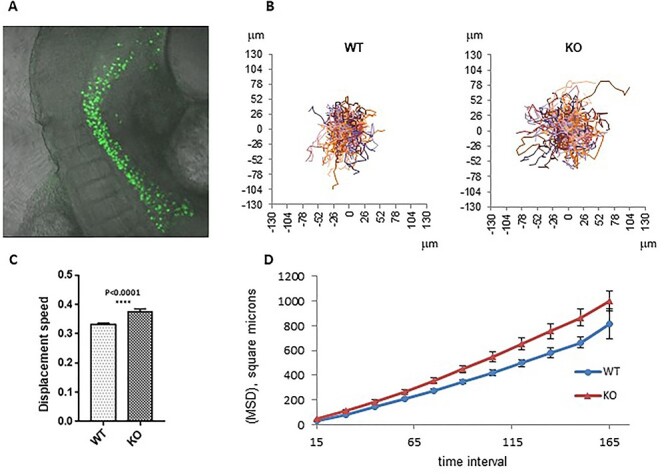
*OG2:Fancg*  ^−/−^ PGCs also exhibited increased cell speed and mean square displacement (MSD) in *ex vivo* E9.5 embryo culture. (**A**) *Ex vivo* migration assay of PGCs. E9.5 embryos were cultured and filmed for 8–12 h, EGFP fluorescence and diffusion light frames were captured every 15 min. (**B**) Movement of *OG2:Fancg*^−/−^ and *OG2:Fancg^+/+^* PGCs shown from the same origin point. (**C**) Average cell speed and (**D**) MSD of tracked PGCs from *ex vivo* culture of E9.5 *OG2:Fancg^+/+^* and *OG2:Fancg*^−/−^ embryos (*P* < 0.005).

Next, we analyzed *in vivo* the motility of PGCs in E9.5 *Fancg*^−/−^ and WT embryos. Using confocal microscopy, time lapse movies of germ cell movements were captured on whole living embryos cultured in basal medium (Fig. 5A). Individual PGCs were tracked over periods of 8–12 h every 15 min (Supplementary Material, video 2). The tracks of the PGCs supported the findings observed *in vitro* (Fig. 5B and Supplementary Material, Fig. S4B and B′), confirming that *Fancg*^−/−^ PGCs explored a larger territory than WT PGCs and displayed an increased motility *in vivo*, as revealed by cell speed and MSD (Fig. 5C and D).

**Figure 6:**
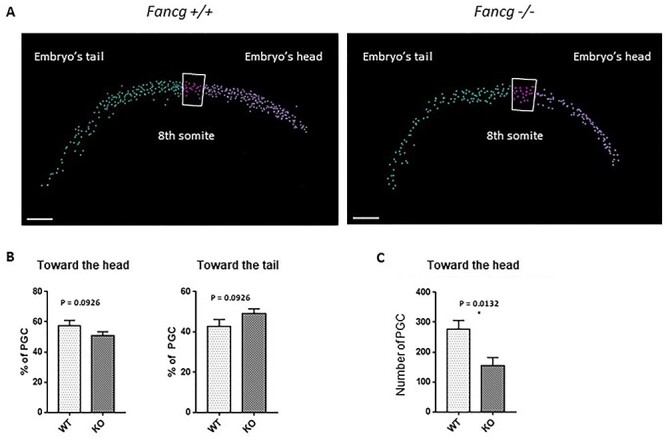
A decreased number of PGCs was found at the front of the migrating wave in E10.5 *OG2:Fancg*^−/−^ embryos. (**A**) Automated cell counting of PGCs using Imaris for confocal microcopy analysis of WT E10.5 cleared embryos using an antibody against EGFP to identify PGCs; the division between the ‘head’ area and the ‘tail’ area was made at the 8th somite (scale = 100 μm). (**B**) Ratios of PGCs found in the head and tail areas over the total number of PGCs in *OG2:Fancg*^−/−^ and *OG2:Fancg^+/+^* embryos (WT *n* = 6, KO *n* = 5). (**C**) Absolute number of PGCs found in the head area in E10.5 *OG2:Fancg*^−/−^ and *OG2:Fancg^+/+^* embryos (WT *n* = 6, KO *n* = 5).

We hypothesized that this abnormally activated random migratory state of *Fancg*^−/−^ PGCs could alter their normal migratory path toward the genital ridges and, therefore, decrease the number of PGCs reaching the gonads at E11.5. Thus, we analyzed E10.5 embryos by confocal microscopy and used somites as morphological landmarks (Fig. 6A). First, the presence of ectopic PGCs did not apparently grossly change in the mutant embryos compared with WT littermates, suggesting that mutation of *Fancg* did not perturb the guided migration to gonads (Fig. 1D). Next, we counted the proportion of PGCs located in the head and tail of the population of migrating PGCs with somite 8 as the boundary line (Fig. 6A). A delay in the migration process should lead to an abnormal proportion of PGCs in the tail of the population. Though the difference was not statistically significant, we observed a slight increase in the proportion of PGCs in the tail in *Fancg*^−/−^ embryos compared with WT embryos mirrored by a decrease in the frequency of *Fancg*^−/−^ PGCs in the front of the migrating wave (Fig. 6B). Remarkably, we observed a decrease in the absolute number of PGCs in the head of the population at the front of the migrating wave in *Fancg*^−/−^ E10.5 embryos, which confirmed a delay in the progression of PGCs to the genital ridges (Fig. 6C). Hence, PGCs attrition in E11.5 embryos may be linked to a loss of PGCs all along the migratory path up to E11.5, likely owing to perturbed random migration and apoptosis in delayed PGCs that did not reach the gonadal ridges at E11.5.

### Abnormal Rac1 activity seems to be involved in the altered migration pattern of Fancg^−/−^ PGCs

**Figure 7:**
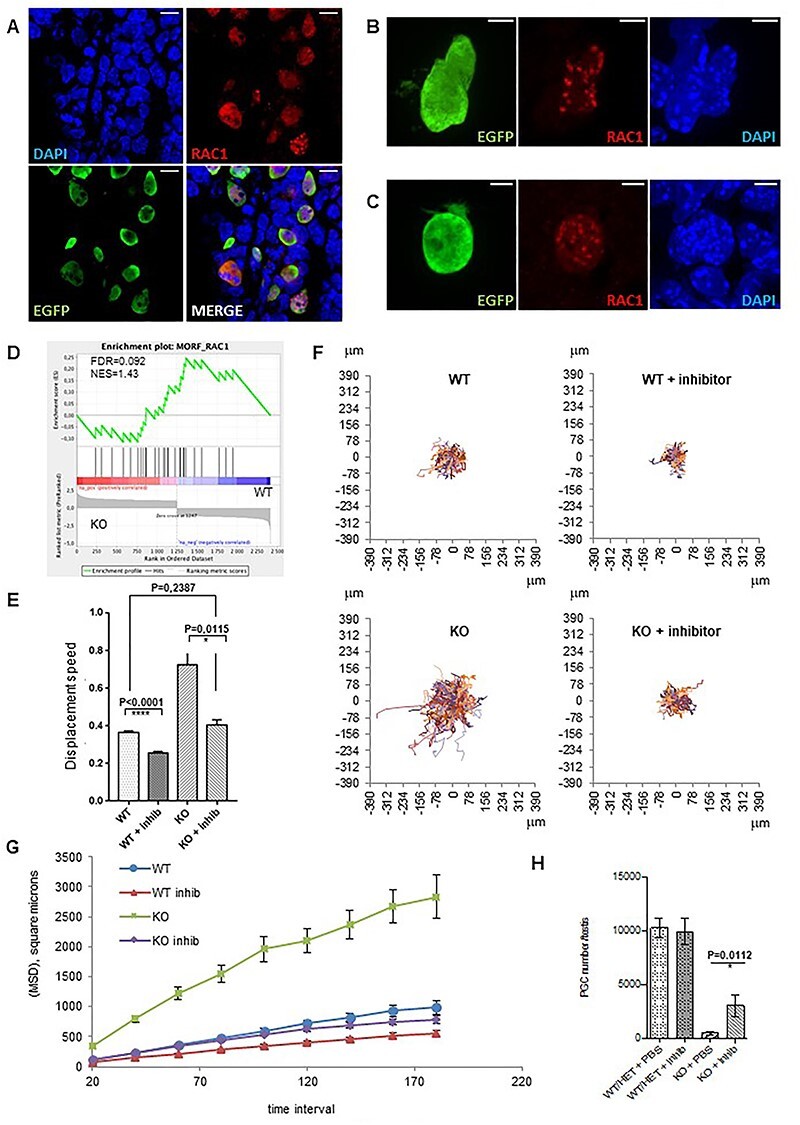
Involvement of Rac1 signaling in abnormal migratory properties and loss of *Fancg*^−/−^ PGCs as shown by recovery of the phenotype in the presence of the Rac1 inhibitor NSC23766. (**A**) Expression of RAC1 in PGCs from E10.5 *OG2:Fancg^+/+^* male embryos. PGCs are labeled with an antibody against EGFP (green), RAC1 (red) and nucleus are stained with DAPI [blue; (scale = 10 μm)]. RAC1 expression in PGCs from E10.5 *OG2:Fancg^+/+^* (**B**) and E10.5 *OG2:Fancg*^−/−^ embryos (**C**) [scale = 5 μm]. (**D**) GSEA showing enrichment plots with gene set enrichment scores (ES) for Rac1. (**E**, **F** and **G**) *In vitro* migration assay of E10.5 PGCs in response to Rac1 inhibitor (acquisition over 7 h, frame every 20 min): average cell speed (E), cell trajectories shown from the same origin (F) and mean square displacement (G) of PGCs from E10.5 *OG2:Fancg*^−/−^ and *OG2:Fancg^+/+^* embryos with or without Rac1 inhibitor [MSD KO vs KO + inhib, *P* < 0.0001; MSD WT vs WT + inhib, *P* < 0.0001]. (**H**) Partial restoration of the loss of PGC in OG2:*Fancg*^−/−^ E14.5 embryos following daily Rac1 inhibitor or PBS administration to pregnant mice from E8.5 to E10.5 (WT/HET + PBS *n* = 12, WT/HET + inhib *n* = 15, KO + PBS *n* = 5, KO + inhib *n* = 4).

Recently, abnormal activity of RAC1 GTPase was reported in FA-deficient head and neck cancer cells and was associated with increased migration and invasive properties (37). RAC1 is involved in the random pattern of migration and cell motility (38). The expression of *Rac1* mRNA was detected in sorted E10.5 PGCs from WT embryos, and we observed an increase of *Rac1* mRNA in PGCs from *Fancg*^−/−^ PGCs when compared with WT PGCs (Supplementary Material, Fig. S5A). In urogenital ridges of E10.5 WT embryos, RAC1 protein was expressed in PGCs, but not in surrounding somatic cells (Fig. 7A). RAC1 was localized in the cytoplasm but also in the nuclei (Fig. 7A and Supplementary Material, Fig. S5B) with a discrete nuclear foci appearance in some PGCs (Fig. 7B). However, we did not observe any difference in the pattern of expression of RAC1 between E10.5 *Fancg*^−/−^ and WT embryos by immunofluorescence (Fig. 7B and C). We performed transcriptomic analysis on sorted PGCs from E13.5 *Fancg*^−/−^ and WT embryos (Supplementary Material, Fig. S6). By focusing the analysis of these transcriptomic data on RAC1 using GSEA, we found that the *Rac1* gene cluster was enriched in E13.5 *Fancg*^−/−^ PGCs compared with WT (Fig. 7D). To test a potential role of abnormal activity of RAC1 in *Fancg*^−/−^ PGCs on motility, we sorted 300 PGCs from E10.5 *Fancg*^−/−^ and WT embryos by flow cytometry and placed the PGCs on inactivated MEFs in the presence of NSC23766, a widely used chemical inhibitor of Rac1 activity. Interestingly, the Rac1 inhibitor completely abrogated the abnormal increase in migratory competence observed in *Fancg*^−/−^ PGCs (Fig. 7E–G), the cell speed and MSD returned to the levels of control WT PGCs. The Rac1 inhibitor also appeared to decrease the directionality of *Fancg*^−/−^ PGCs, with a decay in the directionality ratio, although the direction autocorrelation parameter did not seem to be affected (Supplementary Material, Fig. S4C and C′). We noted that the Rac1 inhibitor also slightly inhibited the random migration of WT PGCs and inhibited *in vitro* the migratory response of WT PGCs to growth factors (Supplementary Material, Fig. S7). Finally, the role of Rac1 was studied *in vivo* during the PGC migratory period*.* The Rac1 inhibitor or PBS as a control were injected daily into pregnant mice from E8.5 to E10.5, and PGCs were counted in E14.5 male fetal gonads. Rac1 inhibitor partially rescued the PGC attrition observed in E14.5 *Fancg*^−/−^ embryos (Fig. 7H) compared with controls. Altogether, these results indicate that the abnormal migratory characteristics observed in *Fancg*^−/−^ PGCs and the subsequent PGC loss in embryonic gonads can be mitigated by modulating the activity of Rac1 using an inhibitor.

## Discussion

The reproductive defect reminiscent of the hypogonadism observed in FA patients is the most consistent phenotype observed in adult FA mouse models. Here, we showed that deletion of the *Fancg* gene*,* a member of the FA core complex, impairs the maintenance of PGCs during fetal life. We observed a defect in the number of PGCs starting at E9.5 in *Fancg*^−/−^ embryos and a strong attrition of PGCs at E11.5, and in male and female E13.5 gonads. The number of PGCs was severely affected when they began to migrate to the gonads at E10.5. A higher rate of PGC apoptosis was observed in E11.5 *Fancg*^−/−^ embryos, which may contribute in part to the critical decrease in the number of PGCs that we observed. The impairment in the development of the germinal lineage at E10.5–11.5 corresponds to the ultimate step of intense migration of PGCs to reach and aggregate in the genital ridges up to E11.5. Our *in vitro* and *in vivo* studies of PGC migration behavior suggested that the random migratory process abnormally activated in *Fancg*^−/−^ PGCs could alter the migration of cells along their normal migratory pathway toward the genital ridges, likely related to an aberrant RAC1 signaling observed in *Fancg*^−/−^ PGCs. Hence, we hypothesized that the progressive attrition of PGCs along the migratory path up to E11.5 could result both from the combination of the proliferation defect and from the perturbed random migration and the subsequent apoptosis in delayed PGCs at E11.5.

PGCs, as other fetal tissue stem cells, must amplify within a short critical window of time during embryonic development. Hence, molecular pathways involved in cell proliferation are critical for fetal stem cell development. The FA pathway is involved in genetic stability during the S and G2/M phases of the cell cycle and particularly contributes to overcoming stalled replication forks during DNA replication (1). The FA pathway also plays a role in mitosis, favoring the resolution of unresolved replication intermediates by the process of anaphase bridge separation (39,40). In FA-deficient embryos, the prevailing model is indeed characterized by an altered response of fetal stem cells to replication stress during the amplification phase and the subsequent proliferation defects, which limits their expansion and results in a diminished pool of fetal stem cells (8,10). A diminished proliferative capacity of *Fancg*^−/−^ PGCs can be intuitively postulated to explain their decreased number in embryos. Proliferative defects during the intense mitotic phase of PGCs at E11.5–E13.5 have been suggested to be implicated in *Fancc*, *Fancl* and *Fancm* germ cell deficits observed in the respective mutant mice. In line with this, we observed in *Fancg*^−/−^ embryos a proliferation deficiency that affected entry into and progression of the S phase of PGCs at E10.5 and E11.5. The recent observation of activation of the p53/p21 axis in human fetal liver samples from FA patients highlights its potential role in the compromised development of fetal hematopoiesis (8). Notably, we also observed exacerbated p53 signaling in E13.5 *Fancg*^−/−^ PGCs using transcriptome analysis. Gene set enrichment analysis revealed that the p53 pathway seemed exacerbated in *Fancg*^−/−^ PGCs compared with WT PGCs (Supplementary Material, Fig. S6), consistent with the proliferative and cell death defects previously observed. Of note, the putative coiled coil domain containing 106 protein (CCDC106) was among the most downregulated proteins in *Fancg*^−/−^ PGCs. CCDC106 is a p53-interacting partner and is described as a novel negative regulator of p53 (41). The ATM-p53-p21 DDR pathway was recently reported to be active in regulating proliferation of male *Fancm*^−/−^ PGCs. However, ATM, p21 or p53 invalidation only partially restored germ cell numbers in *Fancm*^−/−^ neonatal mice (20).

We also observed at E13.5 a heterogeneous pattern of fetal testicular cords with an increased proportion of cords depleted of PGCs in *Fancg*^−/−^ embryos. A diminished proliferative activity of male germ cells once they definitely reside in genital ridges at E11.5 should result in a global and more homogeneous decrease in PGCs that affects all the cords. Hence, the observed mosaic pattern of testicular cords populated with PGCs with cords devoid of PGCs observed at E13.5 suggests that the defect in the mutant mice is linked to impairment in the first phase of PGC development (up to E11.5) during which PGCs proliferate and intensively migrate. Cell migration is a key step in embryonic development, inducing developmental defects in the case of aberrant migration behavior. After their initial migration from the posterior primitive streak to the endoderm at E7.5, PGCs bilaterally migrate between E8.5 and E11.5 via the hindgut endoderm and mesentery to reach the genital ridges. Hence, migration of PGCs through embryonic tissue is complex, requiring fine regulation. The migratory fate of cells results from the coordination of directional migration in response, for example, to extracellular chemotactic signals and random migration allowing the cells to explore their local environment. When we analyzed the tracks of PGCs, we observed *in vitro* and *in vivo* aberrant migration, notably an increased capacity of *Fancg*^−/−^ PGCs to explore their local environment compared with WT PGCs. This suggests that the abnormally activated state of the random migratory abilities of *Fancg*^−/−^ PGCs could alter and delay their travel through the migratory path, causing a decrease in the number of PGCs at the front of the migrating wave at E10.5, as we observed. *Fancg*^−/−^ PGCs seem to progress with less efficiency toward the genital ridges. Of note, the fate of PGCs that do not succeed in reaching the ridges at E11.5 is death (33). In line with this, we observed increased cell death in PGCs at E11.5, contributing to attrition of the pool of PGCs.

Cell migration is a complex process requiring the integration of different signaling pathways (42). Rac1 small GTPase is a regulator of the random pattern of migration and cell motility of several cell types including PGCs in zebrafish embryos (42,43). We found that RAC1 was localized to cytoplasm but also to nuclei in murine PGCs. The RAC1 nucleocytoplasmic shuttling was suggested to play a role in cell migration, the nuclear localization of RAC1 promoting potentially the amoeboid mode of motility in cancer cells (44). PGCs are considered to move actively by amoeboid movements (45), and the nuclear localization of RAC1 that we observed could be related to this. In addition, abnormal activity of Rac1 GTPase was reported in FA-deficient head and neck cancer cells and was associated with increased migration and invasive properties (37). High Rac1 activity was reported to switch cells toward a random migration pattern and impairs directional cell migration in fibroblasts (38,46). Interestingly, the increased migratory behavior of *Fancg*^−/−^ PGCs returned to the level of WT PGCs in the presence of the Rac1 inhibitor. Moreover, the treatment of *Fancg*^−/−^ pregnant mice with Rac1 inhibitor during the migratory stages of PGCs (E8.5–E10.5) partially recovered the dramatic loss of PGCs observed in KO mice at E14.5. Although the mechanisms involved in the connection between RAC1 activity and the FA pathway need further investigation, our results suggest that aberrant migratory behavior linked to abnormal Rac1 activity could contribute to the PGC attrition in E11.5 embryos. Migration is also a key process in the development of fetal hematopoiesis, and aberrant migration behavior could contribute to the fetal HSC defects observed in FA. Deciphering the mechanisms responsible for the developmental defects of the germinal lineage in mice models, especially during fetal development, could benefit our understanding of the pathology seen in FA patients.

## Materials and Methods

### Mice and genotyping

*OG2.Fancg* mice were established by crossing *Fancg^+/−^* mice (31) with *OG2* mice (32). Embryos were produced by heterozygous mating. Males and females were paired overnight and females with a vaginal plug were segregated in the morning (0.5 days post-coïtum). Embryos were genotyped to determine their sex and their *Fancg* status using the following primers: *Fancg* phenotype (wild-type allele, 5′-GGCGACAATGTCCAGCCAGGTCATTCCAGC-3′, 5′-CTTGTAGAGTGAGGAGGAG TTCCCTAAGCC-3′; *Pgk-hygromycin* allele, 5′-GCATCATCGAAATTGCCGTCAACCAA GCTC-3′, 5′-TCGTGCACGCGGATTTCGGCTCCAACAATG-3′), *Sry* (5′-GAGAGCATG GAGGGCCAT-3′, 5′-CCACTCCTCTGTGACACT-3′). Pregnant mice were injected intraperitoneally with 50 mg/kg BrdU and embryos were harvested 2 h after injection. NSC23766 Rac1 inhibitor (3 mg/day/kg) or PBS were intraperitoneally injected into pregnant mice at stages E8.5, E9.5 and E10.5. All animal studies were conducted in accordance with the *Guidelines for the care and use of laboratory animals* of the French Ministry of Agriculture.

### Histology and immunofluorescence

Embryos (E9.5 up to E11.5) and gonads (E13.5) were fixed in 10% neutral-buffered formalin overnight at 4°C, dehydrated, embedded in paraffin wax and cut into 5 μm thick sections. Sections were mounted on slides, dewaxed and rehydrated. For histological analysis, sections were stained with hematoxylin and eosin. For RAC1 expression, embryos (E10.5) were fixed overnight by immersion in 10% neutral-buffered formalin, washed in phosphate-buffered saline (PBS) and then transferred to 20% sucrose overnight. Sections (14 μm) of OCT embedded frozen embryos were cut on a cryostat (Leica, Nanterre, France).

For immunofluorescence, rehydrated tissue or frozen sections were submitted to antigen retrieval by boiling for 20 min in citrate buffer (pH 6). After cooling, sections were permeabilized by incubating the slides in PBS1X-Triton 0.2% for 10 min and then blocked for 30 min in PBS with 5% bovine serum albumin (BSA). Slides were rinsed in PBS1X before incubation overnight at 4°C (1 h RT for frozen sections) with the appropriate primary antibody diluted in PBS1X-BSA0.5% blocking buffer. After rinsing several times, slides were incubated for 1 h with the appropriate secondary antibody and 4,6-diamidino-2-phenylindole (DAPI, 0.4 mg/ml) for DNA staining. Finally, slides were mounted with coverslips in fluorescence mounting medium (S302380, Dako or ProLong Gold, P10144, Life Technologies for Rac1 analysis). To count PGCs, anti-GFP goat polyclonal antibody (1/200e, Abcam, Paris, France, ab6673) and donkey anti-goat Alexa 488 antibody (1/200e, Invitrogen, Thermofisher Scientific, Villebon sur Yvette, France, A11055) were used for secondary staining. To count the number of tubules and to assess the distribution of PGCs in tubules, anti-AMH goat polyclonal antibody (1/200e, Santa Cruz Biotechnology, Heidelberg, Germany, sc-6886) and anti-VASA rabbit polyclonal antibody (1/400e, Abcam, Paris, France, ab13840) followed by donkey anti-goat Alexa 594 antibody (1/200e, Abcam, Paris, France, ab150132) and donkey anti-rabbit Alexa 488 antibody (1/400e, Invitrogen, Thermofisher Scientific, Villebon sur Yvette, France, A21206) for secondary staining were used. To assess mitosis in PGCs, we incubated the sections with anti-Phosphohistone 3 monoclonal mouse antibody (1/300e, Cell Signaling, Ozyme, Saint-Cyr-L'Ecole, France, 9706) and anti-VASA rabbit polyclonal antibody (1/400e, Abcam, Paris, France, ab13840). Sections were then incubated with donkey anti-mouse Alexa 680 secondary antibody (1/300e, Invitrogen, Thermofisher Scientific, Villebon sur Yvette, France, A10038) and donkey anti-rabbit Alexa 488 secondary antibody (1/400e, Invitrogen, Thermofisher Scientific, Villebon sur Yvette, France, A21206). For RAC1 expression, anti-RAC1 rabbit polyclonal antibody (1/100e, Invitrogen, Thermofisher Scientific, Villebon sur Yvette, France, PA1-091X) and anti-GFP goat polyclonal antibody (1/200e, Abcam, Paris, France, ab6673) followed by donkey anti-rabbit Alexa 647 antibody (1/200e, Invitrogen, Thermofisher Scientific, Villebon sur Yvette, France, A31573) and donkey anti-goat Alexa 594 antibody (1/200e, Invitrogen, Thermofisher Scientific, Villebon sur Yvette, France, A11058) for secondary staining were used. TUNEL staining was performed using an ApopTag© Fluorescence *In Situ* Apoptosis Detection Kit (S7110—Millipore, Merck, Fontenay sous Bois, France) according to the manufacturer’s recommendations. Imaging was performed using various fluorescence microscopes: an Olympus AX70 epifluorescence microscope equipped with a CoolSNAP Myo camera Photometrics and Micro-Manager software (version 1.4.16, open source microscopy), a Spinning Disk Confocal CSU-W1 microscope (Yokogawa—GATACA Systems, Massy, France) equipped with Prime 95B camera Photometrics and MetaMorph software (version 7.10.3.279) or also a Nikon A1 laser fluorescence confocal microscope with NIS Elements software (version 4.51, Nikon, Amsterdam Netherlands).

### Flow cytometry and cell sorting

Single cell suspensions were obtained from E13.5 gonads and E11.5, E10.5 or E9.5 embryos after incubation of tissue in a buffer containing PBS1X without Ca^2+^ and Mg^2+^, collagenase (final concentration of 1 mg/ml) and DNase I (final concentration of 0.02 mg/ml) for 5 min at 37°C and dissociation by pipetting 10 times. This step was repeated if needed and cell suspension was filtered at 20 μm and centrifuged for 5 min at 200 g. Cells were then resuspended in PBS1X-BSA0.5% for analysis. To quantify the pool of PGCs, the frequency of the PGC per embryo or testis is reported according to the age of the embryo. In two experiments, BD Trucount™ Tubes method (BD 340334) was used to assess the number of PGCs by flow cytometry (analysis of E9.5 embryos and analysis of E14.5 embryos when NSC23766 Rac1 inhibitor was injected into pregnant mice). BrdU labeling was performed using a APC BrdU Flow Kit (BD Pharmingen, Becton Dickinson, Le Pont de Claix, France) and 7-AAD (7-Aminoactinomycin D) DNA stain according to the manufacturer’s protocol. Single cell suspensions were analyzed using a FACSCalibur™ or LSRII flow cytometer system (BD Biosciences, Becton Dickinson, Le Pont de Claix, France). Data were analyzed with DIVA or FlowJo software. Cell sorting was performed using a FACSAria cytometer (BD Biosciences, Becton Dickinson, Le Pont de Claix, France).

### Transcriptomes

PGCs from E13.5 male embryos were sorted by flow cytometry and used for Affymetrix microarray analysis. Three individual samples of 10 000 PGCs (pooled from at least five embryos for each sample) were processed for WT and KO genotypes. PGCs were directly sorted by flow cytometry in lysis buffer from the RNeasy micro kit (Qiagen, Cortaboeuf, France). The quantity and quality of RNA were analyzed using a 21 000 Bioanalyzer (Agilent Technologies, Les Ulis, France). Only samples with an RNA integrity number (RIN) equal to or above 7 were used for the transcriptomic analysis. RNA samples were then analyzed using an Affymetrix GeneChip™ Mouse gene 2.0 ST Array (Thermofisher Scientific, Villebon sur Yvette, France). Our data were then studied with GSEA and Ingenuity Pathway Analysis software. For *quantitative RT-PCR,* mRNA was prepared using RNeasy® Micro and Mini kits (Qiagen, Cortaboeuf, France). The mRNA was then reverse-transcribed with a Quantitect kit (Qiagen, Cortaboeuf, France). Quantitative RT-PCR was performed using an AB7900 device (Applied Biosystems, Thermofisher Scientific, Villebon sur Yvette, France) with Fast SYBR® Green Master Mix (Applied Biosystems, Thermofisher Scientific, Villebon sur Yvette, France). The primers are listed in Supplementary Material, Table S3.

### Migration assays

Time lapse analysis was performed using a Nikon A1 confocal microscope equipped with a static chamber for cell culture (CO_2_ 5%, O_2_ 18%). After flow sorting, PGCs were placed at 37°C in a static chamber on inactivated MEFs in 96-well plates containing Stemspan™ based medium with 1% ES-cult® fetal bovine serum (Stem Cell Technologies, Saint Egrève, France), 1% antibiotics (Life Technologies, Courtaboeuf, France) and 2% B27 supplement 50X (Life Technologies, Courtaboeuf, France). ckit-ligand at 100 ng/ml (Stem Cell Technologies, Saint Egrève, France), LIF at 1000 u/ml (Merck—Millipore, Merck, Fontenay sous Bois, France), BMP4 at 50 ng/ml (R&D) and SDF-1 at 50 ng/ml (R&D) were added. Rac1 inhibitor, NSC 23766 (Sigma), was used at 50 ng/ml. Cells were recorded 7–12 h every 15 min to study the effect of migration factors and every 20 min to study the effect of the Rac1 inhibitor. For *in vivo* analysis, live E9.5 embryos were maintained in DMEM F12 medium without serum in cell culture inserts (Millicell 0.4 μm) and imaged for 8–12 h every 15 min. We tracked the cells using ImageJ and the MTrackJ add-on (47) and our data were analyzed using the macros developed by Gorelik and Gautreau (34).

### Whole-mount immunostaining, tissue clearing, 3D imaging and analysis

E9.5 and E10.5 embryos were first incubated at room temperature (RT) on a rotating shaker in a solution (PBSGT) of PBS1X containing 0.2% gelatin (Prolabo), 0.5% Triton X-100 (Sigma-Aldrich, L'Isle-d'Abeau Chesnes, France) and 0.1 g thimerosal for 24 h (48). Samples were next transferred to PBSGT containing 0.1% saponin and the primary antibody (1/200e, anti-GFP goat polyclonal antibody, ab6673, Abcam, Paris, France) and rotated at 70 rpm for 1 week at 37°C. This was followed by six washes for 30 min in PBSGT at RT. Next, samples were incubated in secondary antibody (1/200e, donkey anti-goat Alexa 488, A11055, Invitrogen, Thermofisher Scientific, Villebon sur Yvette, France) and TO-PRO3 (1/200e, T3605, Life Technologies, Courtaboeuf, France) diluted in PBSGT overnight at 37°C. After six washes for 30 min in PBSGT at RT, samples were stored at 4°C in PBS until clearing. For clearing, the 3DISCO procedure was used (49). Imaging of transparized embryos was realized using an ultramicroscope (LaVision BioTec, Bielefeld, Germany) and a Nikon A1 confocal microscope. Images and 3D volumes were generated using Imaris x64 software (version 8.2.1, Bitplane AG, Zurich, Switzerland). Stack images were first converted to Imaris files (.ims) using ImarisFileConverter. The embryo was defined as an ROI to mask all background fluorescence outside the embryo. Thus, artifact and non-specific fluorescence surrounding the embryo was segmented and removed. Voxels contained within the created surface (remaining mask) were used for the spot detection function.

### Statistics

All values are means ± SEM. Statistical analysis was performed by Student’s *t* test (GraphPad Prism software). As previously reported (50,51), a two-way analysis of variance with time and condition was used to compare MSD data.

## Accession Number

The GEO accession number for the transcriptomic data reported in this paper is: GSE107504.

## Supplementary Material

Supplementary_data_ddab222

Table_S1_ddab222

Table_S2_ddab222

Table_S3_ddab222
