# Supplementary material for: Abnormal migration behavior linked to Rac1 signaling contributes to primordial germ cell exhaustion in Fanconi anemia pathway-deficient Fancg−/− embryos
Source: Hum Mol Genet. 2021 Aug 9;31(1):97–110. doi: 10.1093/hmg/ddab222 (PMC8682768; doi:10.1093/hmg/ddab222)
Supplement: Supplementary_data_ddab222 [file supplementary_data_ddab222.pdf]

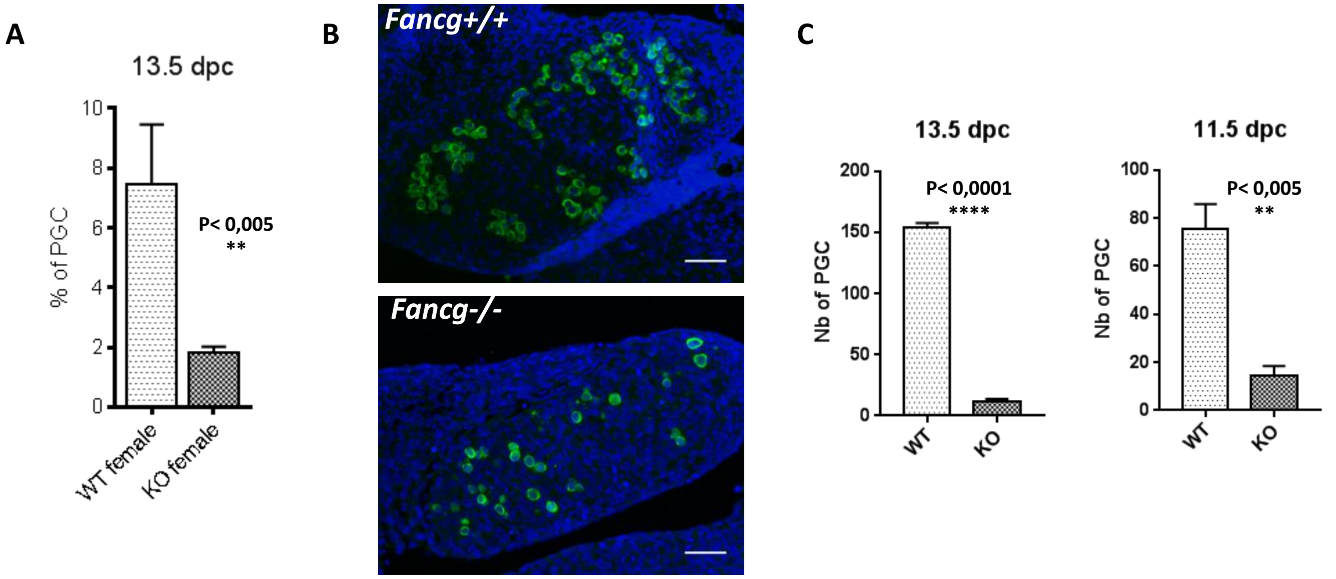

<u>Figure S1</u>: (A) Percentage of PGCs in female E13.5 gonads determined by flow cytometry, showing the significant decrease of PGC in *OG2:Fancg-/-* (n=9) compared to *OG2:Fancg+/+* embryos (n=8). (B) Mouse Vasa Homolog (MVH) immunofluorescence on histological sections of E13.5 male gonad of *Fancg*+/+ and *Fancg-/-* embryos in FVB/NJ genetic background. MVH (green) labels PGCs and nuclei are counterstained with DAPI (blue) (scale=50µm). (C) Counts of PGCs per section of gonad in E13.5 and E11.5 *Fancg*+/+ and *Fancg-/-* embryos, showing the drastic and significant numerical defect in KO gonads (E13.5: WT n=5, KO n=6; E11.5: WT n=5, KO n=3).

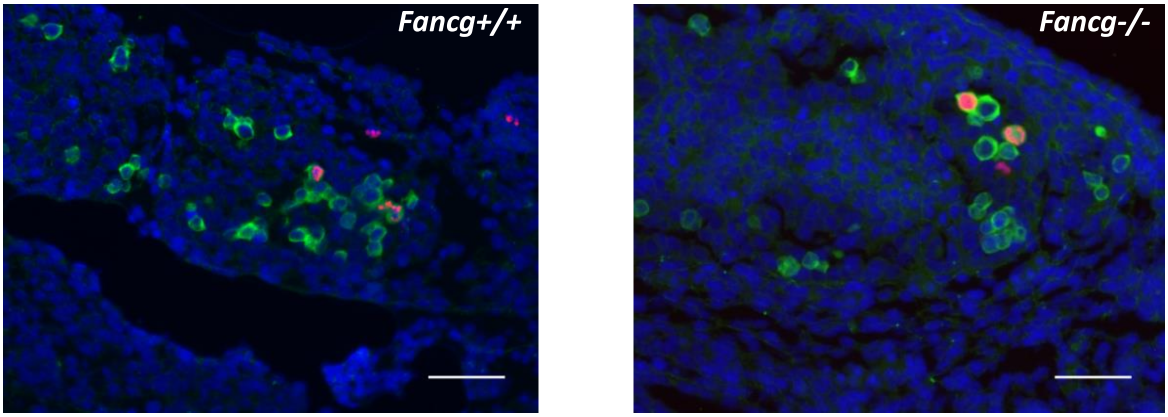

<u>Figure S2</u>: Phosphohistone H3 (PHH3) immunofluorescence on histological sections of male gonads from E13.5 *OG2:Fancg-/-* and *OG2:Fancg+/+* embryos, PHH3 (red), EGFP-positive PGCs (green) and DAPI (blue) (scale=50µm).

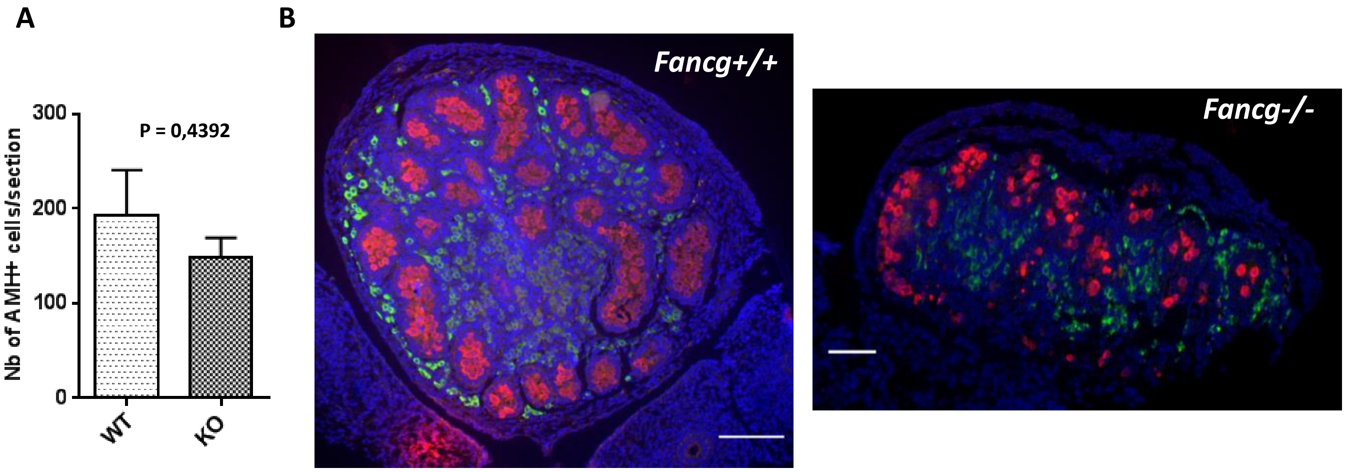

<u>Figure S3</u>: Sertoli and Leydig cells in E13.5 *OG2:Fancg-/-* embryos (A) Count of Sertoli cells in *OG2:Fancg+/+* and *OG2:Fancg-/-* male embryos at E13.5 showing no significant difference (WT n=3, KO n=3). Counts were done from AMH-labelling of histological sections displayed in Fig3A. (B) 3βHSD immunofluorescence on histological sections of male gonads from E13.5 *OG2:Fancg-/-* and *OG2:Fancg+/+* embryos, 3βHSD (green) is a marker of Leydig fetal cells, EGFP-positive PGCs (red) and DAPI (blue) (scale=100µm).

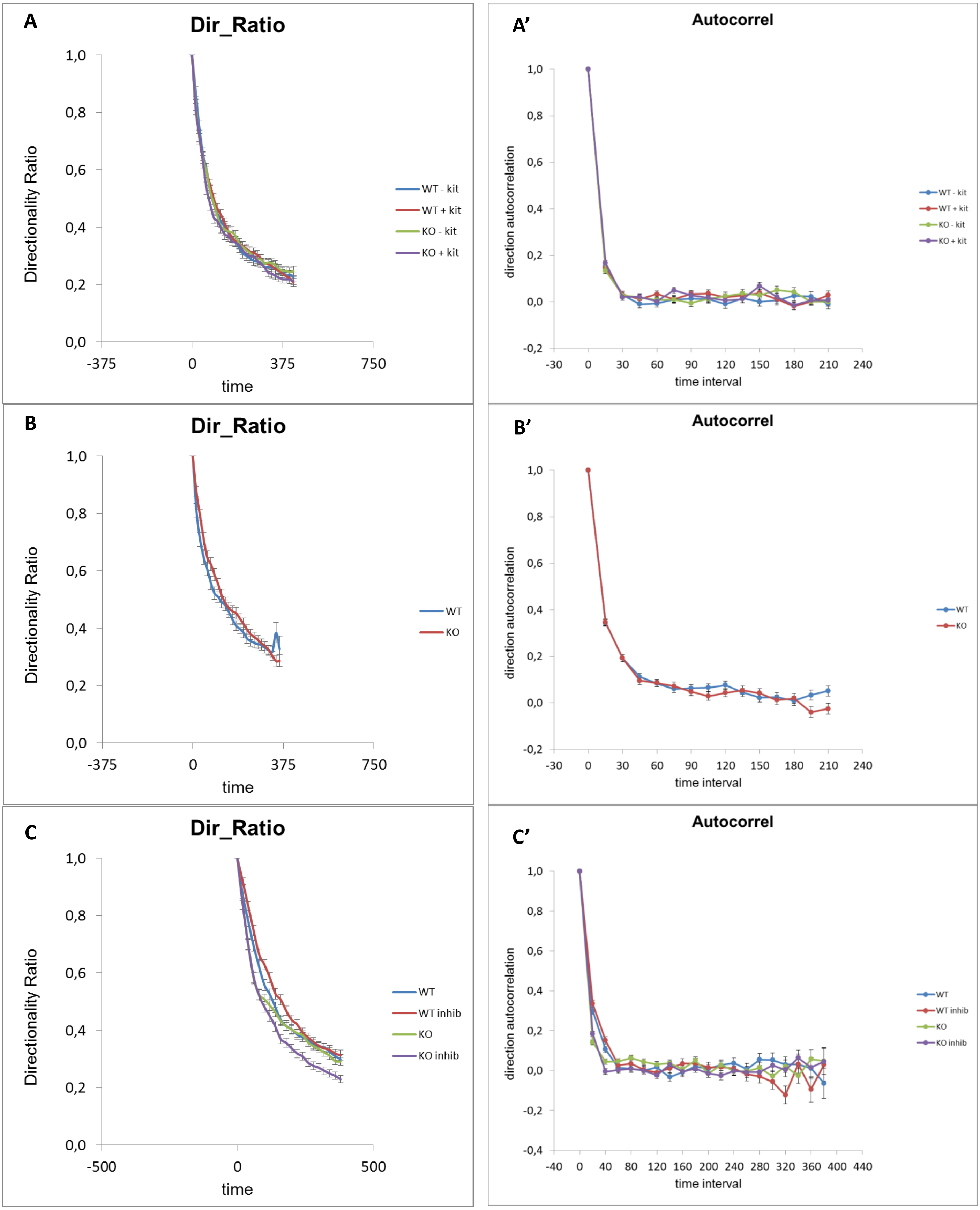

<u>Figure S4</u>: Directional Ratio (A, B, C) and autocorrelation (A', B', C') migration parameters of PGCs from *OG2:Fancg-/-* and *OG2:Fancg+/+* embryos in different conditions.
(A, A') *in vitro* analysis of PGCs sorted from E10.5 embryos in presence or absence of migration factors. (B, B') *ex vivo* analysis of PGCs on culture of E9.5 *OG2:Fancg-/-* and *OG2:Fancg+/+* embryos. (C, C') *in vitro* analysis of PGCs sorted from E10.5 embryos in presence or absence of RAC1 inhibitor NSC23766.

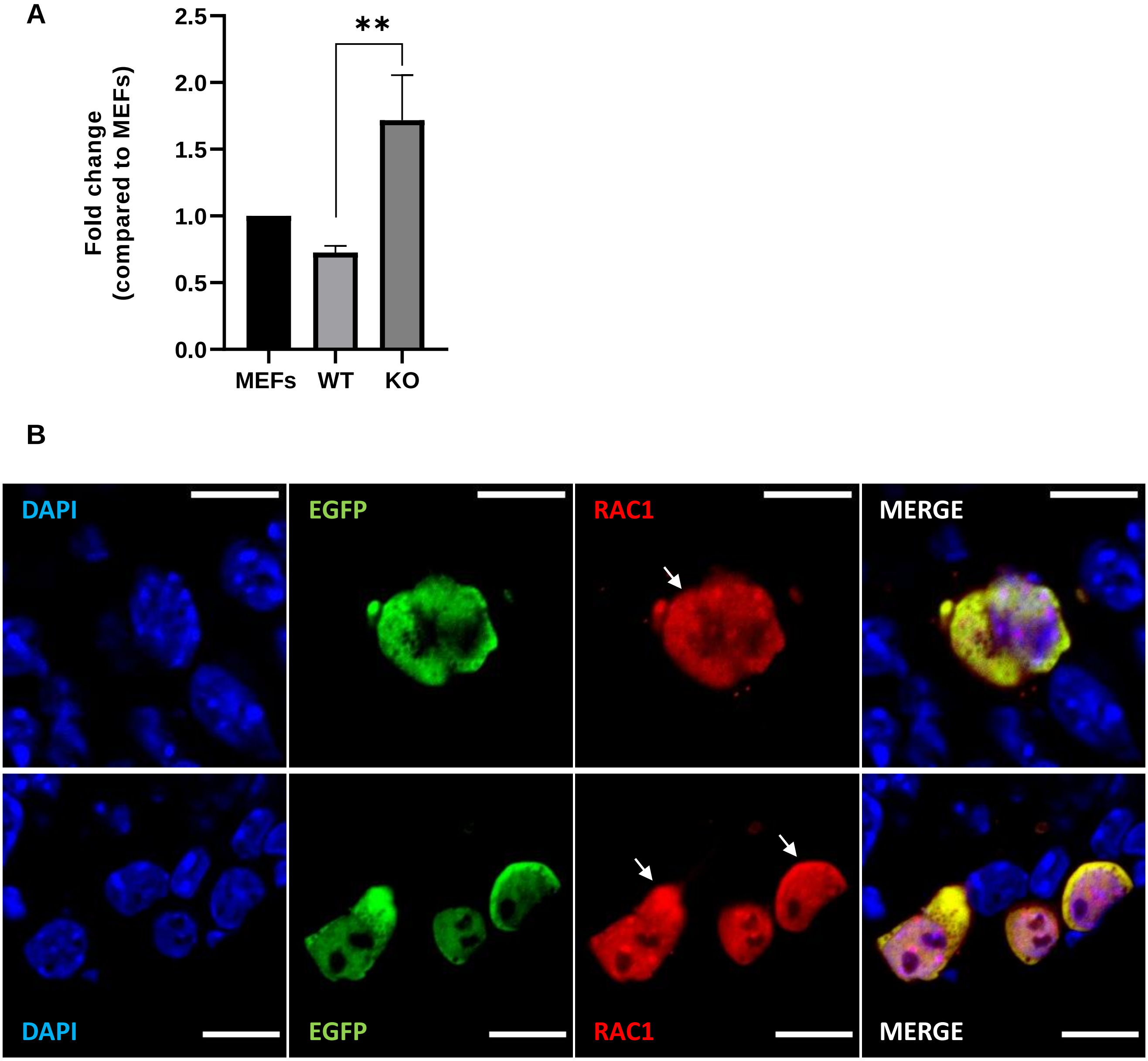

<u>**Figure S5**</u>**:** (A) *Rac1* mRNA expression by RT-qPCR in PGCs from *OG2:Fancg+/+* and *OG2:Fancg-/-* E10.5 embryos (n=3; pool of 3800 PGCs sorted from 3 *Fancg+/+* and pool of 1600 PGCs sorted from 3 *Fancg-/-* embryos), (B) Expression of RAC1 in PGCs from E10.5 *OG2:Fancg+/+* male embryos. PGCs are labeled with an antibody against EGFP (green), RAC1 (red), and nucleus are stained with DAPI (blue) (scale=10 µm). Arrows point to RAC1 expression in the cytoplasm.

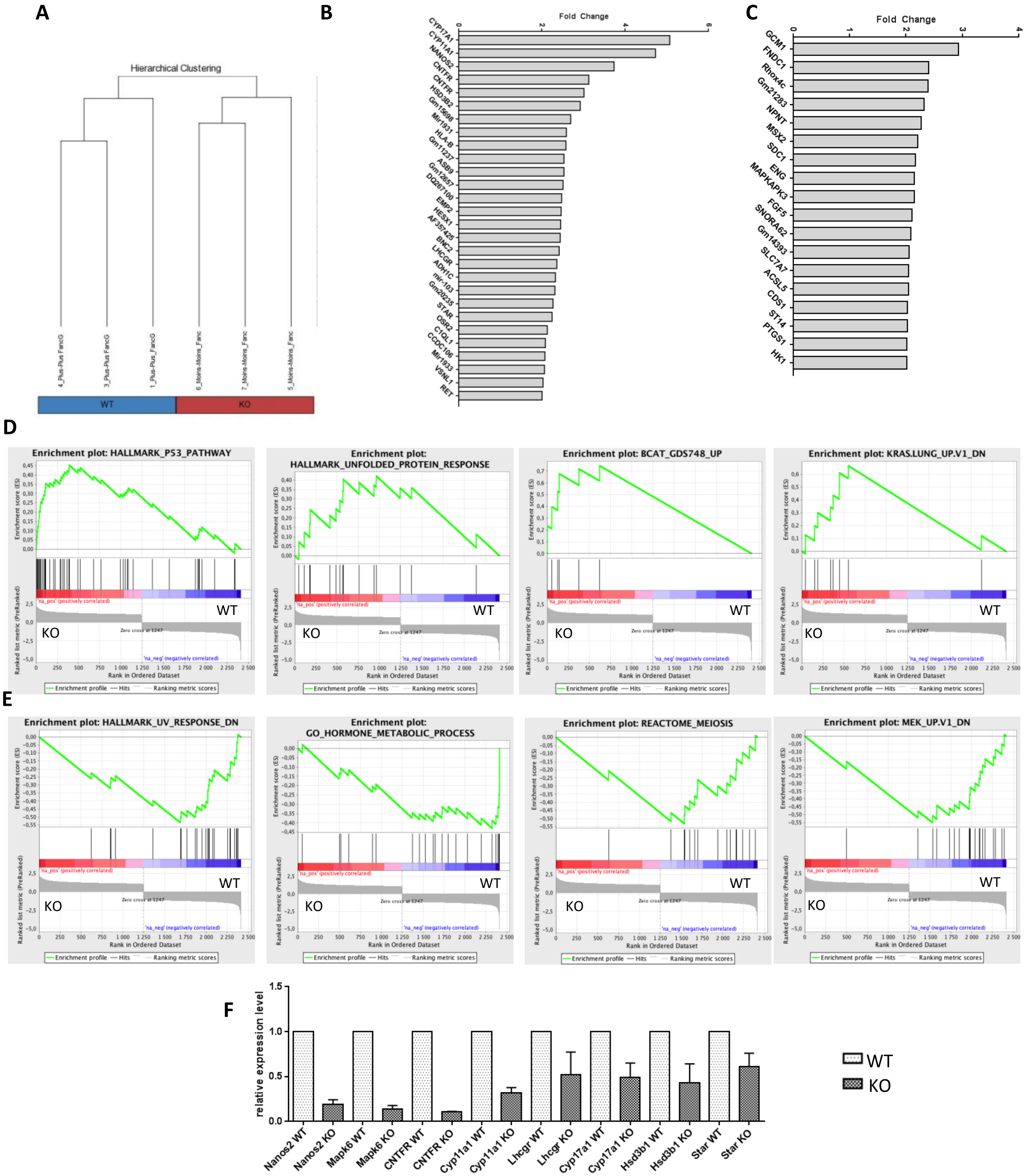

Figure S6: Differential gene expression in *Fancg-/-* PGCs from E13.5 male embryos using microarray analysis. In *Fancg-/-* PGCs, 151 genes were downregulated by more than 1.5-fold (p<0.05), whereas 132 genes were upregulated. (A) Hierarchical clustering of *Fancg-/-* and WT sorted PGCs from E13.5 male embryos (3 pools from at least five embryos for each genotype). (B) Top list of down-regulated genes (fold-change ≤1.5, p<0.05) in *Fancg-/-* PGCs. (C) Top list of up-regulated genes (fold-change ≥ 1.5, p<0.05) in *Fancg-/-* PGCs. See Table S1 and Table S2 for the complete list of genes modulated in *Fancg-/-* PGCs. (D and E) GSEA analysis showing enrichment plots with gene sets enrichment scores (ES) for various gene sets up-regulated (D) and down-regulated (E) in *Fancg-/-* PGCs (FDR≤0.05). NES and nominal P-value (nom. P-value) are shown for each comparison. GSEA revealed that the p53 pathway seemed exacerbated in *Fancg-/-* PGCs compared to WT PGCs, consistent with the proliferative and cell death defects previously observed. Responses to ultraviolet (UV) radiation were found to be downregulated in *Fancg-/-* PGCs by GSEA, likely reflecting impaired DNA damage responses. (F) Validation by RT-qPCR of several genes differentially expressed.

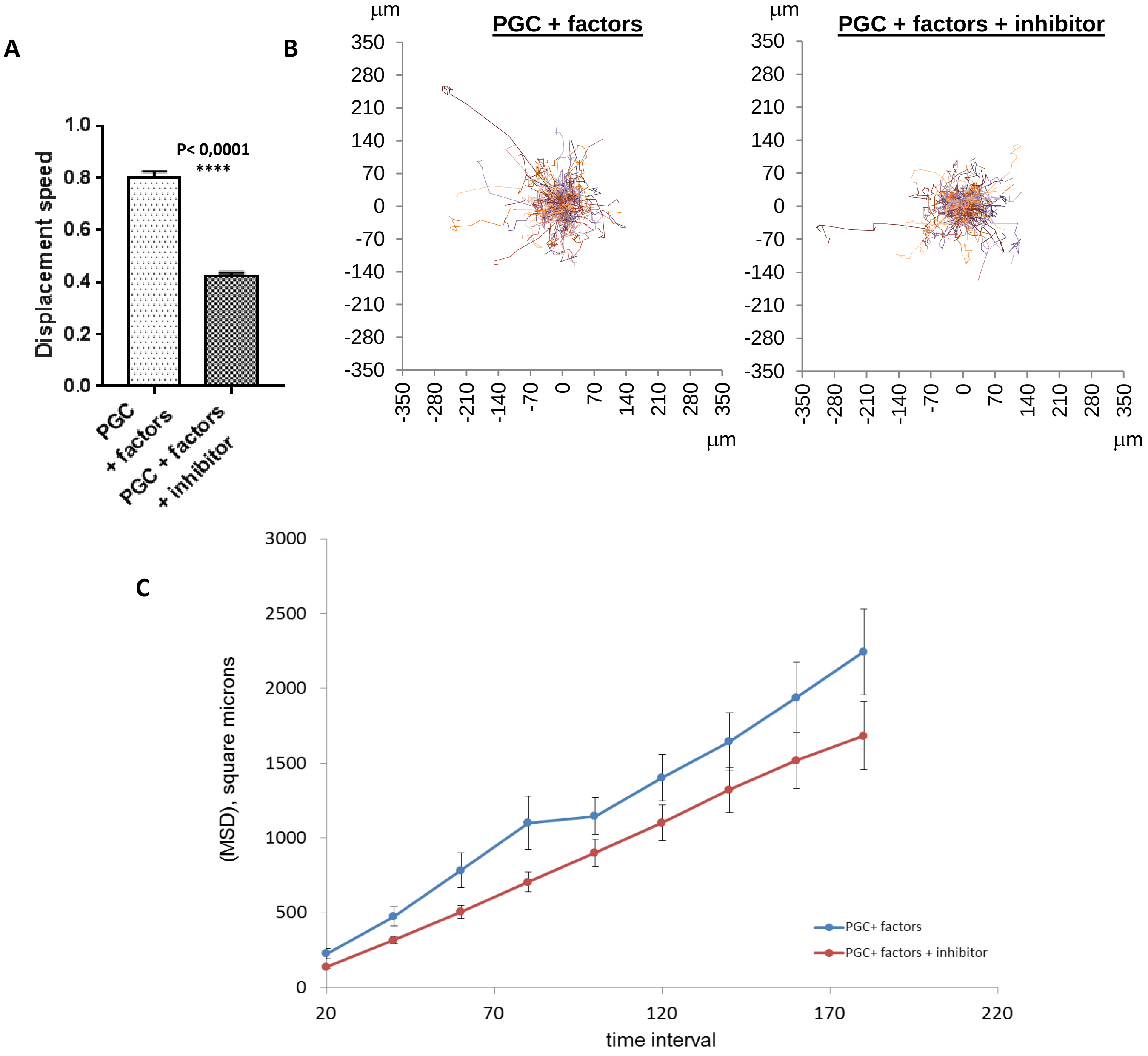

<u>Figure S7</u>: The motility of E10.5 PGCs in response to factor is impaired in presence of the NSC23766 RAC1 inhibitor. Average cell speed (A), cell trajectories brought back to the same origin (B) and mean square displacement (C) of the PGCs from E10.5 *OG2:Fancg+/+* embryos in presence of migration factors (KITL, SDF-1, BMP4, LIF) with or without Rac1 inhibitor NSC23766.

Table S1: List of genes upregulated in E13.5 *Fancg*-/- PGCs (change-fold≥1.5; $p<0.05$)

Table S2: List of genes downregulated in E13.5 *Fancg*-/- PGCs (change-fold≥1.5; $p<0.05$)

Table S3: List of primers for RT-qPCR

Supplementary Video 1: Time lapse video of PGCs sorted from WT E10.5 embryos tracked by EGFP fluorescence and placed on inactivated MEFs. Cell tracking is indicated.

Supplementary Video 2: Time lapse video of EGFP-positive PGCs in E9.5 *Fancg*-/- embryo captured on living whole embryos cultured in basal medium.
